# Prospective Identification and Isolation of Enteric Nervous System Progenitors Using Sox2

**DOI:** 10.1002/stem.557

**Published:** 2010-10-16

**Authors:** Tiffany A Heanue, Vassilis Pachnis

**Affiliations:** Division of Molecular Neurobiology, MRC National Institute for Medical ResearchThe Ridgeway, Mill Hill, London NW7 1AA, United Kingdom

**Keywords:** Enteric nervous system, SOX2, Progenitor cells, Hirschsprung's disease, Stem cell transplantation

## Abstract

The capacity to identify and isolate lineage-specific progenitor cells from developing and mature tissues would enable the development of cell replacement therapies for disease treatment. The enteric nervous system (ENS) regulates important gut functions, including controlling peristaltic muscular contractions, and consists of interconnected ganglia containing neurons and glial cells. Hirschsprung's disease (HSCR), one of the most common and best understood diseases affecting the ENS, is characterized by absence of enteric ganglia from the distal gut due to defects in gut colonization by neural crest progenitor cells and is an excellent candidate for future cell replacement therapies. Our previous microarray experiments identified the neural progenitor and stem cell marker *SRY-related homoebox transcription factor 2* (*Sox2*) as expressed in the embryonic ENS. We now show that *Sox2* is expressed in the ENS from embryonic to adult stages and constitutes a novel marker of ENS progenitor cells and their glial cell derivatives. We also show that *Sox2* expression overlaps significantly with SOX10, a well-established marker of ENS progenitors and enteric glial cells. We have developed a strategy to select cells expressing *Sox2*, by using G418 selection on cultured gut cells derived from *Sox2*^β*geo*/+^ mouse embryos, thus allowing substantial enrichment and expansion of neomycin-resistant *Sox2*-expressing cells. *Sox2*^β*geo*^ cell cultures are enriched for ENS progenitors. Following transplantation into embryonic mouse gut, *Sox2*^β*geo*^ cells migrate, differentiate, and colocalize with the endogenous ENS plexus. Our studies will facilitate development of cell replacement strategies in animal models, critical to develop human cell replacement therapies for HSCR. Stem Cells 2011;29:128–140

## INTRODUCTION

During embryonic development, multipotential progenitor cells give rise to the diverse array of tissue-specific cell types found in adult structures. As development proceeds, however, there is a progressive restriction of cells to particular lineages and within a particular lineage to the differentiated cell types that constitute the mature tissue or organ. Nevertheless, some cells retain multipotential progenitor potential late into embryogenesis, at postnatal stages, and even into adulthood [1]. Cells with such characteristics are highly sought after for a variety of reasons. First, readily accessible multipotential cells allow recapitulation and study of cell lineage progression in culture conditions. Second, the capacity to obtain cells with progenitor cell characteristics offers the potential for stem cell replacement therapies, where progenitor cells can be used to reconstitute cells or tissues that are defective due to injury or disease.

The enteric nervous system (ENS) is the part of the peripheral nervous system (PNS) that controls key aspects of gut function, including peristalsis, the regulation of blood flow, and secretion of water and electrolytes. The mature ENS is composed of neurons and glial cells organized as ganglia within in two concentric rings, the myenteric and submucosal plexus, situated between smooth muscle layers and comprises 1%–5% of cells in the gut [2–4]. One of the most common diseases affecting the ENS is Hirschsprung's disease (HSCR), a congenital disorder occurring in 1:5,000 births and characterized by an absence of enteric ganglia in terminal regions of the gut [2, 5]. Mouse models of HSCR show distal aganglionosis of varying lengths of the gut [2, 5]. Absence of the ENS in distal gut regions has dramatic effects on gut function, causing intestinal obstruction, which can be life-threatening if not treated. Currently, surgical intervention is the routine treatment for infants with HSCR, entailing removal of aganglionic gut regions and rejoining the remaining gut to the anus. However, despite life-saving surgery, such treatment does not necessarily result in complete restoration of normal gut function, and affected individuals often have a lifetime of gastrointestinal problems [6, 7]. Thus, there is considerable interest in the potential of cell replacement therapies to provide complimentary treatment for HSCR. Clinicians and researchers aspire to transplant ENS progenitor cells into aganglionic gut regions and to reconstitute a functional ENS. To realize this aim, much work is underway to identify suitable progenitor cell populations and to devise successful transplantation strategies.

The ENS derives from neural crest cells (NCCs) migrating from the vagal neural tube (enteric neural crest-derived cells [ENCCs]) that colonize the developing gut in a rostral to caudal migratory stream. ENCC progenitors can be identified during embryogenesis by a number of criteria, including being marked by the *Wnt1Cre;Rosa26*^*eYFP*^ NCC lineage marker [8–10] or by expression of SRY-related homoebox transcription factor 10 (*Sox10*; an E-type of high-mobility-group [HMG] box family transcription factors) [11]. Progressive differentiation of progenitors into neurons and glial cells and organization into ganglia occurs within the gut environment [2–4]. Although some progenitor cells give rise to differentiated ENS cells in the gut as early as embryonic day (E) 9.5, when the first HU-expressing enteric neurons are identified [12, 13], progenitor cells persist through perinatal stages, when new neurons and glial cells continue to be born [14–16].

Despite progressive differentiation occurring during embryonic and perinatal stages, in vitro experiments have demonstrated the existence of cells with progenitor potential within embryonic, postnatal, and even adult gut tissues. Cells derived from dissociated gut tissues can be cultivated and expanded, giving rise to neurons and glial cells [2, 17]. These cells not only have proliferation and differentiation potential but are also capable of migrating when transplanted into the developing gut, just like their ENCC precursors [18–20].

To develop cell replacement therapies for HSCR, it is essential to develop robust methods to identify and characterize ENS progenitors and to study their potential following transplantation using animal models. To date, the techniques developed to isolate ENS progenitors involve selection on the basis of cell surface marker expression, culture with factors favoring progenitor cell growth, or selection on the basis of proliferative potential [2, 17]. In the best cases, these techniques can be used to isolate multipotential ENS progenitors, and in some cases, the cells have been shown to function as self-renewing multilineage progenitors (stem cells) [2, 17]. However, some limitations exist to these methods, notably the fact that cells are obtained in small numbers, and that the populations are heterogeneous.

We have previously identified expression of the neural progenitor and stem cell marker SOX2 in the E15.5 mouse ENS [21]. *Sox2* belongs to the B1-type of HMG box family transcription factors and is expressed in neural progenitors of the developing central nervous system (CNS) and in adult CNS stem cells [22, 23]. Identification of *Sox2* expression within the developing ENS, suggested that *Sox2* may also mark progenitors and stem cells within the enteric lineage, a hypothesis we have sought to address in this study. We have characterized SOX2 expression during ENS development, relative to known markers of ENS progenitors. Our results show that SOX2 is expressed in ENS progenitors and glial cells, a profile that largely overlaps with SOX10. Having identified SOX2 as a novel ENS progenitor marker, we have exploited this fact as a means to enrich ENS progenitors from gut-derived cell cultures. We describe an approach to selectively propagate enteric progenitor cells on the basis of *Sox2* expression and demonstrate that cells selected in this way have the capacity to migrate and differentiate following transplantation into the gut environment. Together, our results provide evidence that SOX2 is a new marker of ENS progenitors and that selection of gut-derived cells on the basis of *Sox2* expression enriches cells with progenitor characteristics. These findings offer new tools that will facilitate the study of ENS progenitors/stem cells and provide a readily accessible source of ENS progenitors to enable the development of cell replacement therapies in animal models, both critical components to develop strategies for improved treatment of HSCR patients.

## MATERIALS AND METHODS

### Animals

Wild-type embryonic and postnatal tissues were isolated from Parkes (outbred) mice. *Wnt1Cre;Rosa26*^*eYFP*^, *Sox2*^β*geo*^ and *B6.Rosa26*^*eYFP*^ mice have been described [8–10, 24, 25]. The day of the vaginal plug is considered to be E0.5.

### Immunostaining and β Galactosidase Staining

Tissue sections and acute cultures of gut tissues were generated as described [26, 27]. Peels of postnatal guts were performed as described [26] and fixed flat by pinning. Immunostaining was performed as described [26], with primary antibodies as follows: SOX2 (goat, R&D Systems AF2018, 1:500; rabbit, kindly provided by Michael Wenger (R&D Systems, Abingdon, UK, http://www.rndsystems.com), 1:50), SOX10 (goat, Santa Cruz sc-17342, 1:50; Santa Cruz Biotechnology, Santa Cruz, CA, http://www.scbt.com), TUJ1 (mouse, Covance, MMS-435P, 1:1,000, rabbit, Covance, PRB-435, 1:1,000; Covance, Princeton, NJ, http://www.covance.com), HU (mouse, Invitrogen A-21271, 1:500; Invitrogen, Paisley, UK, http://www.invitrogen.com), green fluorescent protein (GFP; rabbit, Invitrogen A-6455, 1:500; or rat, Nacalai Tesque GF090R, 1:500; Nacalai Tesque, Kyoto, Japan, http://www.nacalai.com), 2H3 (mouse, kindly provided by Marysia Placzek, 1:200), brain fatty acid binding protein (BFABP; rabbit, Millipore AB9558, 1:500; Millipore, Billerica, MA, http://www.billerica.com), S100 (rabbit, DAKO Z0311, 1:500), glial fibrillary acidic protein (GFAP) (rabbit, DAKO Z0344, 1:500), Ki67 (mouse, BD Pharmingen, 550609, 1:50; Pharmingen, San Diego, CA, http://www.bd.com) and with fluorescently conjugated secondary antibodies as follows: Cy3 and Cy5 (Jackson, West Grove, PA, http://www.jacksonimmuno.com, AlexaFluor 488 and 568, Invitrogen, 1:500). For β galactosidase staining, tissues, tissue sections, or cells were fixed for 10 minutes and stained as described [28].

### *Sox2*^β*geo*^ Cell Culture

Midgut and hindgut tissue was isolated from embryonic and postnatal *Sox2*^β*geo*/+^ or *Sox2*^β*geo*/+^; *B6.Rosa26*^*eYFP*/+^ mice. Dissociation of gut tissues was performed as described [26]. After 1 day in culture, media was changed, and on day 2, cells were passaged as described [26]. On day 3 of culture, the media was replaced with media containing G418 (Genetecin, Invitrogen, 200 μg/ml). Expansion of cells followed protocols for generation of neurosphere-like bodies (NLBs) [26], although cells were maintained as adherent cultures. Plates were passaged while the cells were adherent, prior to NLB formation, and continuously maintained in media containing G418. Cells were maintained in culture with periodic passaging for at least 2 months and are termed *Sox2*^β*geo*^ cells. Withdrawal experiments involved a change to media without G418 for 4 days of further culture.

### Ex Vivo Cell Transplantation

Recipient guts were isolated from Parkes embryos. *Sox2*^β*geo*^ cells were transplanted into the stomach or the cecum region of E11.5 guts using pulled capillary micropipets [20]. E11.5 hindgut segments and E12.5 distal hindgut segments were dissected to represent uncolonized regions of gut [29], and cells were transplanted into the proximal end. Guts and gut segments containing transplanted cells were cultured in free-floating culture for 4 days as described [20], then fixed for immunostaining or β galactosidase staining.

## RESULTS

### SOX2 Is Expressed in Migratory ENCCs and Expression Persists at Adult Stages

*Sox2* was identified as an ENS-expressed gene in a microarray screen to identify novel markers of the developing ENS [21]. To gain insight into the possible roles of *Sox2* during ENS development, we analyzed the spatial and temporal expression profile of SOX2 during key phases of ENS development.

**Figure 1:**
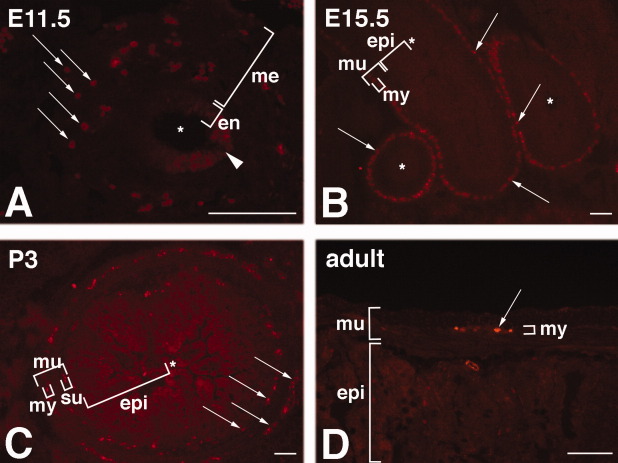
SRY-related homoebox transcription factor 2 (SOX2) is expressed within the mouse enteric nervous system over a broad developmental time frame. Immunohistochemistry was conducted using an anti-SOX2 antibody on cross sections through the developing **(A, B)** and postnatal **(C, D)** gut. Landmarks of the radially organized gut cross sections are noted for reference, and asterisks denote the lumen of the gut tube. **(A)**: At E11.5, SOX2 expression is seen in the foregut endoderm (en; arrowhead) and in a punctate pattern consistent with expression in enteric neural crest-derived cells within the mesenchymal layer (me; arrows). **(B)**: At E15.5, SOX2 expression in the midgut is observed in rings of cells corresponding to the location of the myenteric plexus (my) within the muscular layers (mu; arrows). **(C)**: At P3, SOX2 expression in the midgut is observed in two rings, consistent with expression in the myenteric and submucosal (su) plexus layers within the muscular layers (arrows). **(D)**: At adult stages, SOX2 expression can be seen within clusters formed between midgut muscle layers corresponding to the location of ganglia of the mature myenteric plexus (arrow). The gut epithelial layer is denoted as epi. Scale bar = 100 μm.

At E11.5, when ENCCs are migrating along the gut wall, SOX2 expression is observed in both the foregut endoderm (Fig. 1A, arrowhead), consistent with previous reports [24], and within a scattered population of cells within the gut mesenchyme (Fig. 1A, arrows). As development proceeds, ENCCs differentiate and become organized as a plexus of ganglia. At E15.5, SOX2 expression is observed in a ring of cells corresponding to the location of the myenteric plexus (Fig. 1B, arrows). The submucosal plexus forms at postnatal stages, and at P3, SOX2 is expressed in two concentric rings of cells, consistent with expression in the two plexus layers (Fig. 1C, arrows). Within the adult gut, SOX2 is expressed in clusters of cells in sites corresponding to the location of enteric ganglia (Fig. 1D, arrows). These results suggest that SOX2 is expressed within the ENS over a broad developmental time frame.

**Figure 2:**
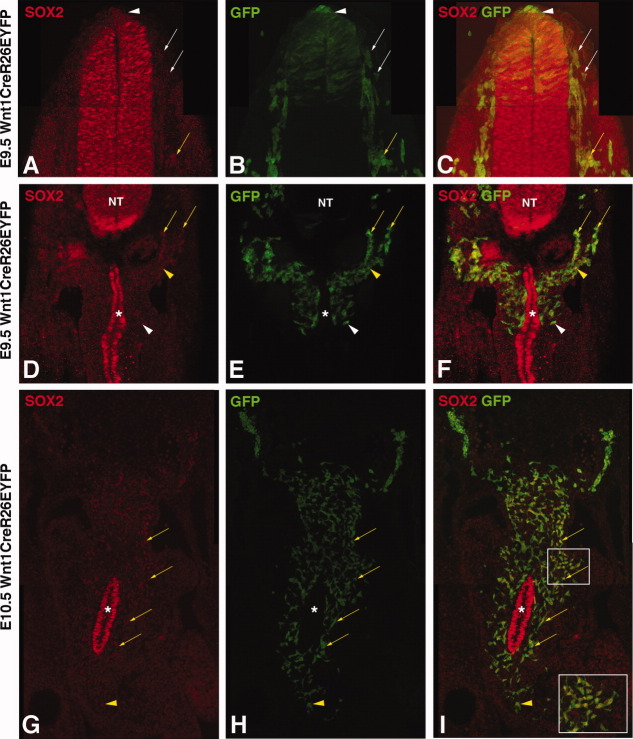
SOX2 expression is downregulated in early migratory enteric neural crest-derived cells (ENCCs), but SOX2 is expressed in ENCCs within the gut. Immunohistochemistry was conducted on sections of E9.5 **(A–F)** and E10.5 **(G–I)** embryos from the *Wnt1Cre;Rosa26*^*eYFP*^ background, which express enhanced yellow fluorescent protein in all neural crest-derived cells (NCCs), using antibodies for SOX2 **(A, D, G)** and GFP **(B, E, H)**. Merged images are shown **(C, F, I)**. **(A–C)**: SOX2 is expressed throughout the NT. NCCs undergoing initial migration from the neural tube downregulate SOX2 (white arrows). Faint antibody staining is detected in isolated NCCs adjacent to the ventral neural tube (yellow arrow). **(D–F)**: At positions where ENCCs are observed colonizing the foregut (white arrowhead; foregut endoderm indicated by asterisk), SOX2 expression is not observed in ENCCs. SOX2 expression is observed within small numbers of NCCs at positions between the ventral neural tube and the dorsal aorta (da; between yellow arrows and yellow arrowhead). **(G–I)**: At E10.5, when ENCCs are migrating extensively through the gut, SOX2 is expressed in ENCCs at all positions within the gut (yellow arrows, foregut indicated by asterisk). Coexpression of nuclear and cytoplasmic GFP (green) and nuclear SOX2 (red) is evident as green cells containing yellow/orange nuclei (see inset in **[I]**, which corresponds to boxed region). Abbreviations: GFP, green fluorescent protein; NT, neural tube; SOX2, SRY-related homoebox transcription factor 2.

To verify that SOX2 is expressed within ENCCs, and to characterize the onset of SOX2 within this population, we analyzed SOX2 expression within the NCC lineage using embryos from the *Wnt1Cre;Rosa26*^*eYFP*^ background, which express enhanced yellow fluorescent protein (EYFP) in all neural crest-derived cells. At E9.5, SOX2 is expressed throughout the neural tube (NT; Fig. 2A, 2D) and foregut endoderm (asterisk, Fig. 2D) [24]. Expression of SOX2 is downregulated in NCCs undergoing their initial migration from the NT (Fig. 2A–2C, white arrows), consistent with previous findings [30]. We find that SOX2 is first expressed within the NCC lineage at E9.5 (Fig. 2). A small number of individual SOX2-expressing NCCs are observed adjacent to the ventral NT and dorsal aorta (Fig. 2A–2C, yellow arrow; Fig. 2D–2F, between yellow arrows and yellow arrowhead), which may represent precursors of the sympathetic lineage [31]. However, NCCs invading the foregut do not express SOX2 (Fig. 2D–2F, white arrowhead). By E10.5, however, SOX2 is expressed in ENCCs at all positions within the gut, including the cells at the front of migration (Fig. 2G–2I, arrows).

### SOX2 Is Expressed in ENS Progenitors

**Figure 3:**
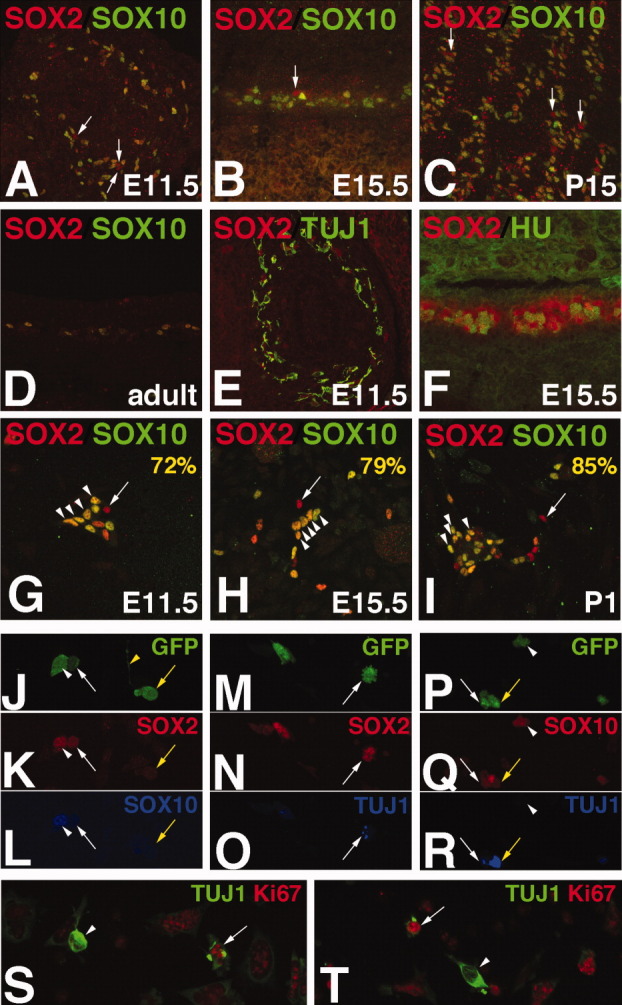
SOX2 is expressed in all SOX10-expressing enteric neural crest-derived cells and SOX2 expression is excluded from differentiated neurons. **(A–F)**: Immunohistochemistry comparisons of SOX2 expression with expression of SOX10 **(A–D)** and enteric neuron markers TUJ1 **(E)** and HU **(F)** on cross sections through the E11.5 midgut **(A, E)**, E15.5 midgut **(B, F)** and adult midgut **(D)**, and peel preparations of P15 midgut outer muscle layers **(C)**. **(A–D)**: SOX2 and SOX10 expression is largely overlapping over a broad developmental time course. However, cells expressing only SOX2 can be identified at embryonic and early postnatal stages (**[A–C]**, arrows). **(E, F)**: SOX2 expression is excluded from cells expressing TUJ1 and HU. **(G–T)**: Immunostaining on acute cultures of gut tissues from E11.5 **(G, J–T)**, E15.5 **(H)**, and P1 **(I)** wild-type animals **(G–I, S, T)** or *Wnt1Cre;Rosa26*^*eYFP*^ embryos **(J–R)**. **(G–I)**: Comparison of SOX2 and SOX10 expression reveals that the majority of SOX2-expressing cells express SOX10 (**[G–I]**, arrowheads; 72% at E11.5, 79% at E15.5, and 85% at P1) but that a distinct population expressing only SOX2 is evident (SOX2^+^ SOX10^−^; **[G–I]**, arrows). **(J–L)**: Analysis of acute cultures from *Wnt1Cre;Rosa26*^*eYFP*^ shows that all SOX2-expressing cells express green fluorescent protein (GFP) and are therefore derived from the neural crest (white arrow, white arrowhead). SOX2-expressing cells either coexpress SOX10 (white arrowhead) or do not (white arrow). GFP-expressing SOX2^−^SOX10^−^ cells (yellow arrowhead) have projections characteristic of differentiated neurons (yellow arrowhead). **(M–O)**: SOX2 is expressed in neural crest-derived cells displaying punctate TUJ1 staining (arrow) but not in cells displaying a normal TUJ1 pattern (**[J, K]**, yellow arrow). **(P–R)**: SOX10 is expressed neither in cells displaying a punctate pattern of TUJ1 staining (white arrow) nor in cells displaying normal uniform pattern of TUJ1 staining (yellow arrow). SOX10 is expressed in cells that do not express TUJ1 (arrowhead). **(S, T)**: Cells exhibiting a punctate pattern of TUJ1 staining also express Ki67 (arrows), and are therefore still within active phases of the cell cycle, whereas cells displaying a normal TUJ1 pattern do not express Ki67 (arrowheads), and are therefore postmitotic. Abbreviations: GFP, green fluorescent protein; SOX2, SRY-related homoebox transcription factor 2; SOX10, SRY-related homoebox transcription factor 10.

To further characterize SOX2 expression in the ENS, we compared expression of SOX2 with that of known ENS markers. SOX10 represents an established marker of ENS progenitors and enteric glial cells [11, 26, 32]. During progressive stages of ENS development, all SOX10-expressing cells coexpress SOX2 (Fig. 3A–3C), indicating that SOX2 is expressed in ENS progenitors. We also identify a small population of cells that express SOX2 but are negative for SOX10 (SOX2^+^ SOX10^−^; arrows in Fig. 3A–3C). By adult stages, SOX2 and SOX10 show completely overlapping expression, and contrary to findings at embryonic stages, we were unable to identify any SOX2^+^ SOX10^−^ cells (Fig. 3D). Comparison of SOX2 expression with that of the neural markers HU and TUJ1 shows that, like SOX10, SOX2 is not expressed within neural populations of the ENS (Fig. 3E, 3F) [26]. These data reveal that SOX2 is expressed in ENS progenitors and offer suggestive evidence that SOX2 is also expressed within enteric glial lineages.

**Figure 4:**
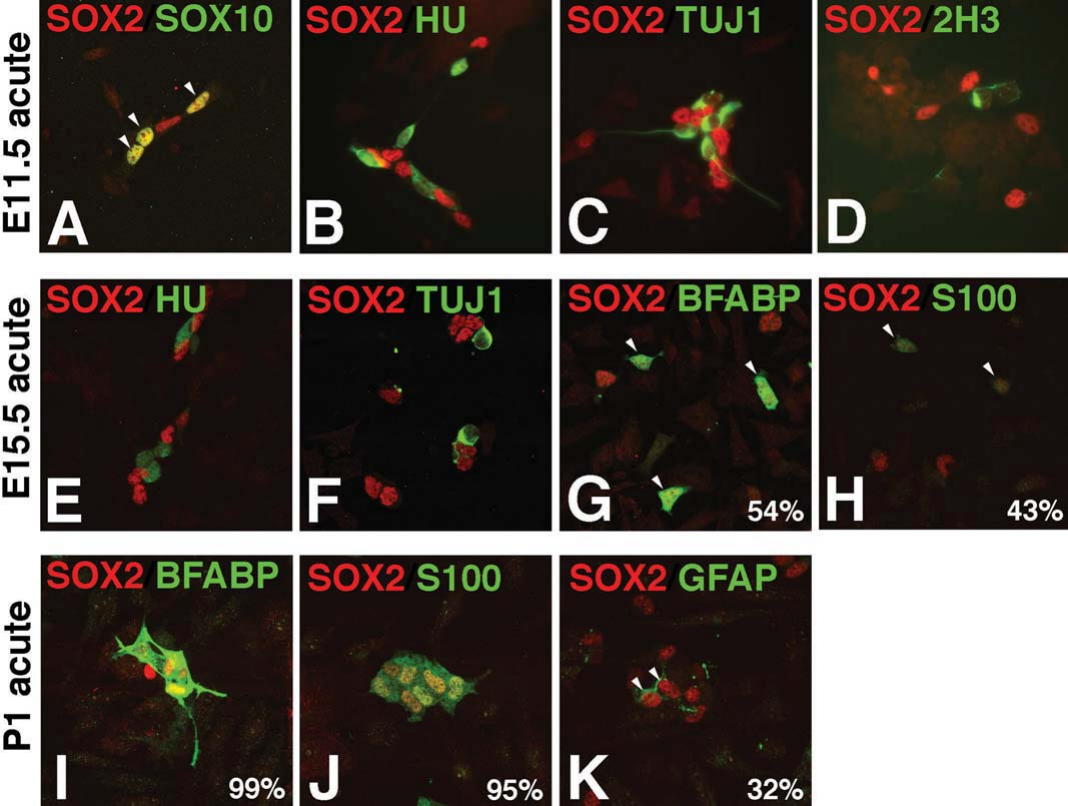
SOX2 is expressed in enteric nervous system (ENS) progenitors and glial cells. Immunostaining on acute cultures from E11.5 **(A–D)**, E15.5 **(E–H)**, and P1 **(I–K)** gut tissues using markers of ENS progenitors (SOX10, **[A]**), neural differentiation (HU, TUJ1, 2H3, **[B–F]**), and glial cell differentiation (BFABP, S100, GFAP, **[G–K]**). **(A)**: SOX10 is expressed in the majority of SOX2-expressing cells at E11.5. Expression of neural markers HU, TUJ1, and 2H3 are excluded from SOX2-expressing cells at E11.5 **(B–D)** and E15.5 **(E, F)**. **(G, H)**: At E15.5, a large proportion of SOX2-expressing cells coexpress BFABP (54%, arrowheads **[G]**) and S100 (43%, arrowheads **[H]**). **(I–K)**: At P1, almost all SOX2^+^ cells express markers of progressive glial cell differentiation, BFABP (99%, **[I]**), S100 (95%, **[J]**), and GFAP (32%, **[K]**, arrowheads). Abbreviations: BFABP, brain fatty acid binding protein; GFAP, glial fibrillary acidic protein; SOX2, SRY-related homoebox transcription factor 2; SOX10, SRY-related homoebox transcription factor 10.

To further characterize the SOX2^+^ SOX10^−^ population, we analyzed expression in short-term (acute) cultures of embryonic and postnatal tissues. SOX2 and SOX10 are coexpressed in the majority of cells, that is, at E11.5, 72% of SOX2-expressing cells coexpress SOX10 (Fig. 3G, also Fig. 4A). As development proceeds, the proportion of SOX2/SOX10 coexpressing cells increases from 79% at E15.5 to 85% at P1. (Fig. 3H, 3I). Accordingly, the SOX2^+^ SOX10^−^ population decreases during development (from 28% to 21% to 15% at E11.5, E15.5, and P1, respectively).

We verified that all SOX2-expressing cells are derived from NCCs using acute cultures established from E11.5 *Wnt1Cre;Rosa26*^*eYFP*^ embryonic midgut and hindgut and show that all SOX2-expressing cells are EYFP^+^ (data not shown and Fig. 3J, 3K, 3M, 3N). This analysis further identified three distinct populations of neural crest-derived cells, that is, cells coexpressing SOX2 and SOX10 (SOX2^+^SOX10^+^, 68.1% of GFP^+^ cells; Fig. 3J–3L, white arrowhead), cells that express only SOX2 (SOX2^+^ SOX10^−^, 15.6% of GFP^+^ cells; Fig. 3J–3L, white arrow), and cells that express neither SOX2 nor SOX10 (SOX2^−^ SOX10^−^, 16.3% of GFP^+^ cells; Fig. 3J–3L, yellow arrow). SOX2^+^SOX10^+^ cells show no TUJ1 staining and represent ENS progenitors (arrowhead in Fig. 3P, 3R; Fig. 4C and data not shown). SOX2^+^ SOX10^−^ cells show punctate TUJ1 expression (Fig. 3M–3O, white arrow). The fact that SOX10 is not expressed in such cells (Fig. 3P–3R, white arrow) demonstrates that these cells are not progenitor cells or glial cells [11, 26, 32] and indicates that they belong to the neural lineage, consistent with the presence of TUJ1 expression. However, because a normal TUJ1 pattern is not observed, we conclude that such cells are undifferentiated cells of the neural lineage or cells undergoing early neuronal differentiation. Consistent with this conclusion, we show that cells exhibiting punctate TUJ1 (SOX2^+^ SOX10^−^) are Ki67^+^ (Fig. 3S, 3T, arrows), indicating that they are still within active phases of the cell cycle. SOX2^−^ SOX10^−^ cells have differentiated as neurons and have neuronal morphology and display a normal profile of TUJ1 expression (yellow arrow in Figs. 3J–3L, 3P–3R, 4C). Cells expressing this normal TUJ1 profile are Ki67^−^ (Fig. 3S, 3T; arrowheads), reflecting the fact that they are postmitotic. Taken together, these results indicate that SOX2 is expressed in ENS progenitors and undifferentiated or early differentiating cells of the neural lineage, but it is excluded from differentiated neurons.

### SOX2 Is Expressed in Glial Cells

The coexpression of SOX2 and SOX10 during later embryonic stages (Fig. 3H, 3I), and the expression of SOX2 in non-neural ENCCs (Fig. 3E, 3F), suggested that SOX2 is also expressed in glial cells. We tested this idea directly by comparing SOX2 expression with markers of neural versus glial cell differentiation. SOX2 expression is absent from cells expressing the pan-neural markers HU, TUJ1, and neurofilament (2H3; Fig. 4B–4F). In contrast, all cells expressing glial cell markers, such as BFABP, S100, and GFAP, express SOX2 (arrowheads in Fig. 4G–4K; supporting information Fig. 1).

Glial cell differentiation can be tracked through progressive acquisition of glial cell markers, the first being BFABP, followed by S100b, and last GFAP [16]. At E15.5, 54% of SOX2-expressing cells express the early glial cell marker BFABP. S100 identifies further differentiated glial cells and marks 43% of SOX2-expressing cells. At P0, 99% and 95% of SOX2-expressing cells express BFABP and S100, respectively, and 32% of SOX2-expressing cells coexpress the mature glial cell marker GFAP. Taken together, our results show that SOX2 is expressed in all glial cells and identifies both early differentiating and mature glial cells.

### ENCCs Can Be Selected on the Basis of *Sox2* Expression

**Figure 5:**
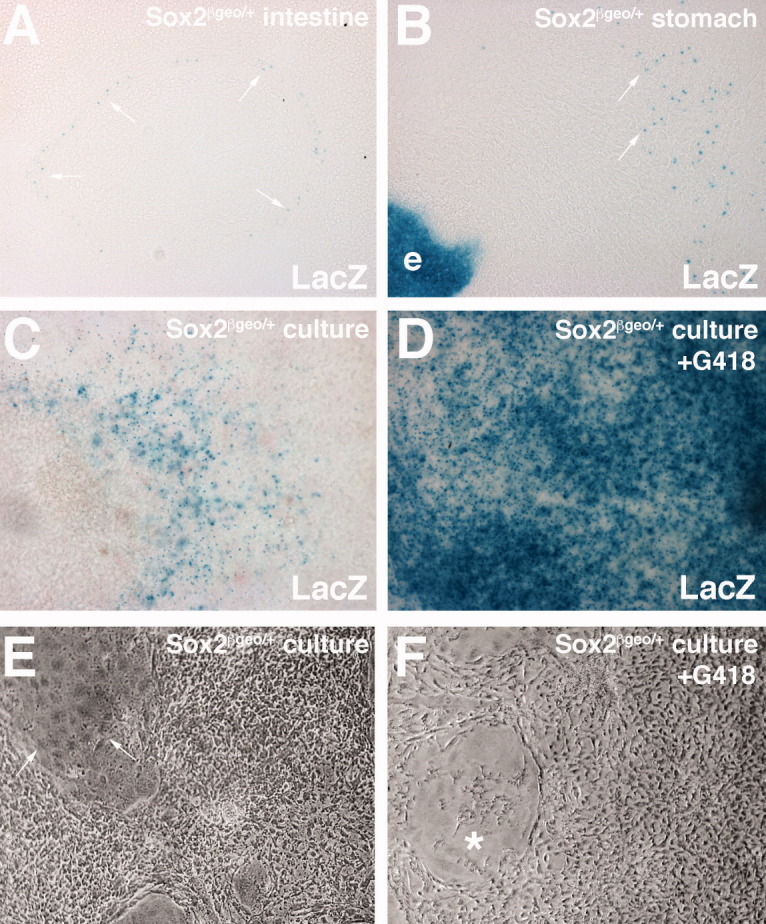
*Sox2*^β*geo*/+^ mice express the βgeo transgene within the enteric nervous system (ENS), thus allowing selection of *Sox2*-expressing cells in culture. **(A, B)**: β galactosidase staining on sections of E14.5 intestine (midgut; **[A]**) and stomach (foregut; **[B]**) from *Sox2*^β*geo*/+^ embryos reveals expression of the βgeo transgene (blue cells) within normal sites of *Sox2* expression, the ENS (arrows), and the foregut endoderm (e). **(C)**: β galactosidase staining on primary cultures established from E14.5 *Sox2*^β*geo*/+^ embryos reveals the presence of cells expressing the βgeo transgene (blue cells). **(D)**: Primary cultures from E14.5 *Sox2*^β*geo*/+^ midgut and hindgut tissue treated with G418 display a massive enrichment of cells expressing the βgeo transgene. **(E, F)**: Bright field images of primary cultures established from E14.5 *Sox2*^β*geo*/+^ reveals the presence of large flat cells that correspond to smooth muscle cells (**[E]**, arrows), which are absent in cultures treated with G418 **(F)**. Asterisk indicates voids present in G418-treated cultures that are likely to represent sites where G418 eliminated non-*Sox2*-expressing smooth muscle cells **(F)**. LacZ indicates β galactosidase staining.

Our results demonstrate that SOX2 represents a novel marker of enteric progenitor cells within the embryonic and/or postnatal ENS. Therefore, we reasoned that we might be able to identify and select for ENS progenitors on the basis of SOX2 expression. Toward this end, we analyzed the *Sox2*^β*geo*^ line, a knock-in of a β galactosidase/neomycin-resistance fusion gene (β*geo*) into the *Sox2* locus [24], for potential use to allow selection of *Sox2*-expressing ENS progenitors. LacZ staining on sections of E14.5 *Sox2*^β*geo*/+^ embryos demonstrates that the β*geo* transgene is expressed within the ENS. Punctate LacZ staining is detected in regions corresponding to the myenteric plexus (Fig. 5A, 5B, arrows) and that are distinct from the previously described foregut endoderm expression of SOX2 [24] (Fig. 5B, e).

We harnessed the expression of the β*geo* transgene within *Sox2*-expressing ENS population to place midgut and hindgut tissues derived from *Sox2*^β*geo*/+^ embryos under G418 selection, thereby eliminating any non-*Sox2*-expressing cells but allowing survival and expansion of neomycin-resistant ENCC-derived *Sox2*-expressing cells. We established primary gut cultures from E14.5 *Sox2*^β*geo*/+^ embryos using protocols that promote the expansion of ENCCs relative to other gut-derived cell types [26]. Following 2 days of culture, G418 was added to the culture media. At this point, *Sox2*-expressing cells comprise 3.3% of the cells in the culture (data not shown). After 4 days, cells were analyzed by LacZ staining, which revealed dramatic enrichment of βgal-expressing cells relative to untreated control cultures (compare Fig. 5D with Fig. 5C). Bright field examination of these cultures shows that untreated control cultures contain clusters of large smooth muscle cells (arrows in Fig. 5E). In cultures treated with G418, smooth muscle cells are absent, and the gaps observed in the cultures (asterisk in Fig. 5F) likely correspond to sites where non-*Sox2*-expressing smooth muscle cells were eliminated by addition of G418. After 7 days, untreated cultures contain 8.4% *Sox2*-expressing cells, whereas G418-treated cultures contain 96.1% *Sox2*-expressing cells (data not shown). Similar results were obtained by culturing *Sox2*^β*geo*/+^ tissues derived from E11.5 embryos and from peels of the myenteric layer of postnatal and adult guts (data not shown). These data suggest that G418 selection of *Sox2*^β*geo*/+^ tissue cultures leads to massive enrichment of *Sox2*-expressing cells and loss of non-ENCC cell types, such as smooth muscle cells. Moreover, *Sox2*^β*geo*^ seven cells can be maintained and expanded in culture for at least 2 months (data not shown), suggesting the potential for long-term culture and generation of large numbers of *Sox2*-expressing cells.

**Figure 6:**
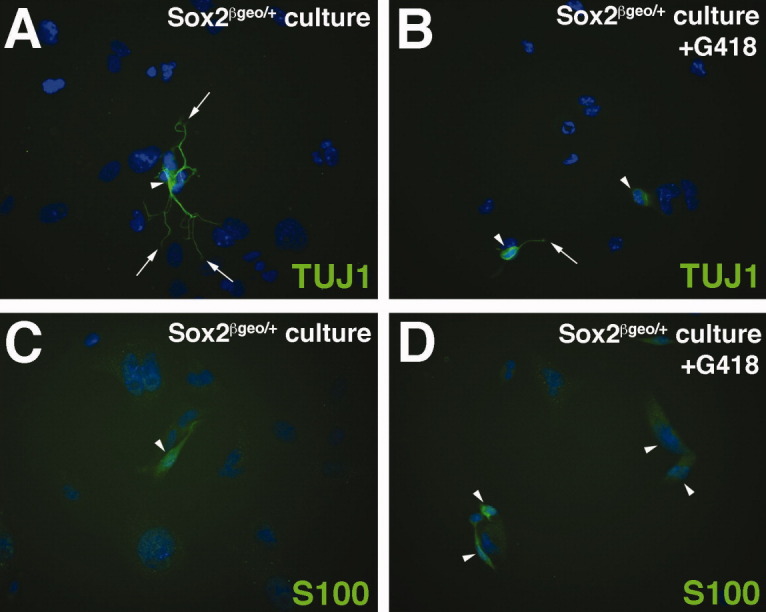
Sox2^βgeo^ cell cultures are enriched for enteric neural crest-derived cell and glial cells and do not contain mature neurons. Immunostaining of primary cultures derived from E14.5 *Sox2*^β*geo*/+^ midgut and hindgut tissues after 4 days without treatment **(A, C)** or treated with G418 **(B, D)** using markers of neural (TUJ1, **[A, B]**) and glial cell development (S100, **[C, D]**). **(A)**: Fully differentiated TUJ1-expressing neurons (arrowhead), which are characterized by strong staining and multiple elaborated cell processes (arrows), are frequently observed in untreated cultures derived from *Sox2*^β*geo*/+^ embryos. **(B)**: Following treatment with G418, cells that express TUJ1 do so at low levels (arrowheads) and do not contain elaborated processes characteristic of differentiated neurons but contain only short processes (arrow). **(C, D)**: The proportion of S100-expressing cells (arrowheads) is increased following treatment with G418.

Analysis of ENCC marker gene expression within control and G418-treated *Sox2*^β*geo*/+^ embryonic gut cultures reveals that ENCC-derived cell types are dramatically enriched through this selection protocol. At the start of culture, SOX10-expressing cells represent 2% of cultured cells, whereas TUJ1-expressing cells represent 1.7% of cultured cells and S100-expressing cells are undetected (data not shown and supporting information Fig. 2A, 2B). After just 2 days of culture, G418 selection quickly leads to large increases in the proportion of SOX10-expressing cells within the culture (41.2% vs. 9.3% in untreated controls; data not shown), and after 7 days of selection, 75.1% of cells express SOX10 versus 5.2% in untreated controls (data not shown and supporting information Fig. 2C, 2E). On 7 days of G418 selection, 14.4% of cells are found to express TUJ1 versus 1.7% in untreated control cultures (data not shown). Consistent with the fact that fully differentiated neurons do not express SOX2, no neurons expressing high levels of TUJ1 are found in G418-treated cultures, although these are evident in control cultures (Fig. 6A, 6B, arrows; supporting information Fig. 2D, 2F). Presumably, following loss of SOX2 expression on complete neuronal differentiation (transition from low to high levels of TUJ1), neurons are eliminated. On removal of G418 selection, however, cells are capable of differentiating as neurons expressing high levels of TUJ1 and possessing long neuronal processes (supporting information Fig. 2G).

The proportion of glial cells also increases on selection; following 2 days of G418 selection, 16.3% of cells express S100 versus 6.3% in untreated control cultures (Fig. 6C, 6D; data not shown), and after 7 days of culture, 23.1% express S100 versus 4.3% in controls (data not shown and supporting information Fig. 2C, 2E). It is significant that the culture conditions used here favor progenitor cells over differentiated cell types [26], so although glial cells express SOX2 and have the potential to constitute a large portion of the selected population, the culture conditions used here keep glial cell numbers relatively low.

Together, these data show that we have established a protocol for selecting and expanding *Sox2*-expressing ENCCs in culture (hereafter referred to as *Sox2*^β*geo*^ cells). Using our protocol, cultures are enriched for SOX2^+^SOX10^+^ ENCCs, which represent both ENS progenitors and glial cells, and show a striking absence of differentiated neurons in the population.

### *Sox2*^β*geo*^ Cells Possess Migratory and Differentiation Potential

The development of a protocol for generating enriched populations of ENS progenitors lends itself well for modeling strategies for stem cell replacement therapies for diseases of the ENS, such as HSCR. It is therefore of considerable interest to further characterize the properties of these cells following transplantation into animal models.

**Figure 7:**
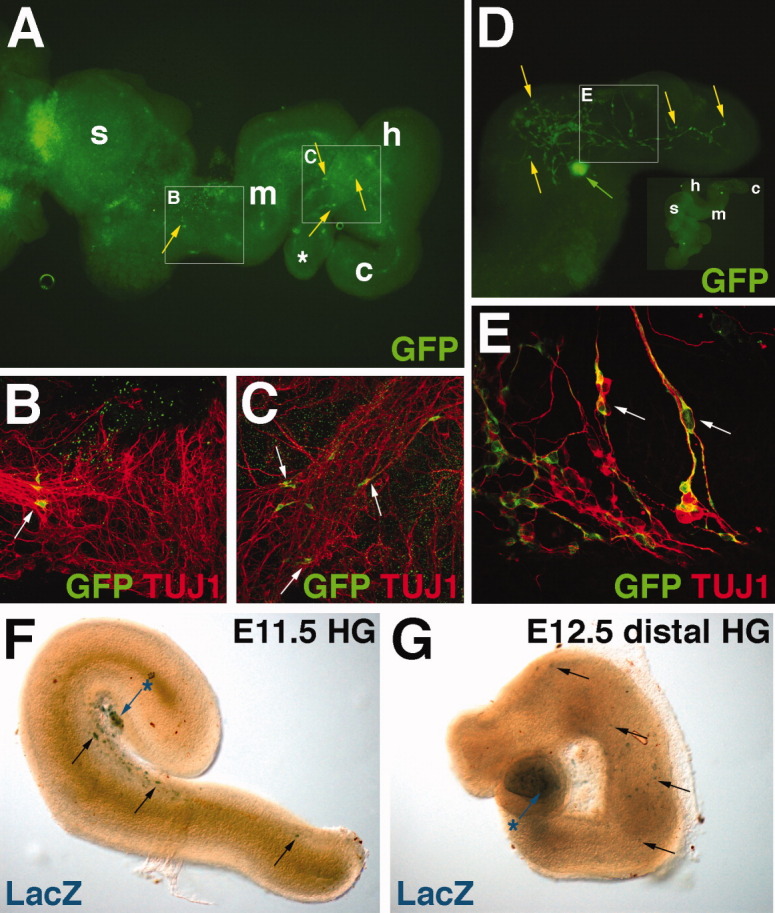
*Sox2*^β*geo*^ cells possess migratory and differentiation potential. **(A–E)**: Immunostaining of cultured wild-type guts 4 days after transplantation of enhanced yellow fluorescent protein (EYFP)-expressing *Sox2*^β*geo*^ cells into the E11.5 gut, using GFP to detect transplanted cells and TUJ1 to detect differentiated neurons **(B, C, E)**. **(F, G)**: LacZ detection of *Sox2*^β*geo*^ cells transplanted into uncolonized regions of the E11.5 hindgut and E12.5 distal hindgut. **(A)**: Following transplantation of a small number of cells (less than 50) into the stomach (s) of an explanted gut, EYFP-expressing cells (in green) can be observed in the midgut (m; yellow arrows in area bounded by box B) and distal hindgut (h; yellow arrows in area bounded by box C). **(B, C)**: GFP and TUJ1 immunostaining in higher magnification view of box B and C in **(A)** shows transplanted GFP^+^ *Sox2*^β*geo*^ cells among endogenous TUJ1-expressing enteric neurons (in red). **(D)**: The site of transplantation of hundreds of cells into the enteric nervous system-sparse cecum region (c), between the midgut (m) and the hindgut (h; see inset **E**) is identified by intense GFP expression (green arrow). Transplanted EYFP-expressing *Sox2*^β*geo*^ cells have migrated away from the site of transplantation (yellow arrows). **(E)**: GFP and TUJ1 immunostaining in higher magnification view of box E in **(D)** shows clearly that transplanted EYFP-expressing *Sox2*^β*geo*^ cells express TUJ1 (arrows) and possess long processes. The transplanted TUJ1-expressing cells (in yellow) are found in close association with endogenous TUJ1-expressing neurons (in red). **(F, G)**: Following transplantation of *Sox2*^β*geo*^ cells into the proximal region of the E11.5 HG **(F)** or E12.5 distal hindgut (**[G]**, blue arrows, asterisks), cells migrate to the distal end of the gut segment (black arrows). Abbreviations: GFP, green fluorescent protein; HG, hindgut.

We tested the migratory and differentiation properties of *Sox2*^β*geo*^ cells following transplantation into the murine gut. To distinguish transplanted cells from the endogenous ENS, we have generated *Sox2*^β*geo*^ cells from animals that are expressing EYFP in all cells (*Sox2*^β*geo*/+^; B6.Rosa26^eYFP/+^), so that transplanted cells can be identified on the basis of their fluorescence. A small number of *Sox2*^β*geo*^ cells were grafted into the stomach region of explanted E11.5 guts. After 4 days in culture, guts were examined to identify EYFP^+^ *Sox2*^β*geo*^ cells. EYFP^+^ cells can be observed in the midgut (Fig. 7A, 7B) and distal hindgut (Fig. 7A, 7C), indicating that *Sox2*^β*geo*^ cells are capable of migrating over long distances through the developing gut. Transplantation of hundreds of EYFP^+^ *Sox2*^β*geo*^ cells into the relatively ENS-sparse cecum region, between the midgut and hindgut, shows clearly that transplanted *Sox2*^β*geo*^ cells express TUJ1 and have long projections characteristic of fully differentiated neurons (Fig. 7D, 7E). Moreover, transplanted *Sox2*^β*geo*^ cells are closely associated with endogenous, non-EYFP-expressing neurons (Fig. 7D, 7E). Finally, we have tested the capacity of *Sox2*^β*geo*^ cells to migrate within uncolonized gut regions of E11.5 hindgut and E12.5 distal hindgut [29], an established model of aganglionic gut regions [18, 33], and demonstrate that *Sox2*^β*geo*^ cells are capable of migrating from the site of transplantation (Fig. 7F, 7G, asterisks) along the length of these gut regions (Fig. 7F, 7G, arrows). Taken together, these results demonstrate that *Sox2*^β*geo*^ cells possess key properties of ENS progenitors, such as migratory potential and differentiation potential and are capable of colonizing aganglionic gut regions.

## DISCUSSION

We have established SOX2 as a novel marker of ENSs progenitor cells and their glial cell derivatives. The expression profile of SOX2 shows high degrees of similarity to that of SOX10, a well-established enteric progenitor and glial cell marker. Furthermore, we have demonstrated that cells selected on the basis of *Sox2* expression (*Sox2*^β*geo*^ cells) have characteristics of ENS progenitors and are capable of migrating and differentiating following transplantation into a gut environment. Therefore, *Sox2*^β*geo*^ cells will be useful tools in efforts to model stem cell replacement therapies.

### Coexpression of SOX2 and SOX10 in ENS Progenitors and Glial Cells

The profile of expression we describe for SOX2 in ENS progenitors and enteric glial cells is largely overlapping with that described for SOX10, with two notable differences. First, SOX10 is expressed in NCCs as they delaminate from the NT and continues to be expressed in migratory NCCs during migration into the developing gut. In contrast, SOX2 expression, which is found throughout the NT, is downregulated among delaminating and early migrating NCCs. However, on reaching the developing gut, SOX2 is upregulated and from this point onward, SOX2 and SOX10 show extensive overlap in expression. A second notable exception is that SOX2 is transiently expressed in undifferentiated cells of the neural lineage or early differentiating neurons, whereas SOX10 is not. So, although SOX2 and SOX10 are both downregulated on neural differentiation, SOX10 is downregulated more rapidly. Consistent with this finding, in the spinal cord, expression of the SOX B1 group proteins (SOX1, SOX2, SOX3) is only gradually extinguished as neural differentiation proceeds [34], whereas SOX10 is more tightly downregulated, such that SOX10 is only transiently coexpressed in a few cells that express early differentiation markers such as MASH1 [35]. Whether isolated expression of SOX2 simply identifies early commitment to neuronal differentiation within the ENS or has an additional role in regulating this process warrants further investigation. The expression profiles of SOX2 and SOX10 in the developing ENS glial lineages are indistinguishable. Thus, despite the extensive overlap between SOX2 and SOX10 expression, the existence of two significant differences in their expression profiles suggests differences in the regulation of these two related proteins. Whether differences in regulation may reflect differences in function remains to be determined.

At postnatal stages, the vast majority of SOX2^+^ cells are glial cells. However, we also identify a small population of postnatal SOX2^+^ cells that do not express glial markers (representing 1% of the SOX2^+^ population at P1). These cells may represent early differentiating neurons or ENS progenitors that are known to exist within these tissues [26]. Interestingly, studies show that ENCCs that express neither neural nor glial cell markers (PGP9.5 and S100b, respectively) comprise 1.7% of the P0 ileum and 4.7% of the P0 colon and are proposed to represent ENS progenitors [16]. Given the similarities in numbers, we suggest that SOX2^+^ cells in the postnatal gut that do not express glial cell markers may correspond to ENS progenitors. This population may be the source of multilineage progenitors found within cultures of postnatal gut tissues [26] and could also constitute the source of neurogenic progenitors identified in the adult [36].

### Profiles of SOX2 Expression Vary Between Regions of the Nervous System

Comparing the expression profile of SOX2 in the ENS with the expression profile of SOX2 in other nervous system regions reveals some clear similarities and some striking differences. SOX2 has been studied most extensively within the CNS, where it is expressed in early neuronal progenitors, in neural stem cells, and in small numbers of mature neurons, but only rarely in CNS glial cells [37, 38]. In the dorsal root ganglia (DRG) of the PNS, SOX2 is expressed on arrival of migrating NCCs to the DRG [39], turned off in differentiated neurons but maintained in glial cells [39, 40]. Thus, our observations of SOX2 expression within developing ENS are more similar to SOX2 expression during DRG development, rather than in CNS development. However, a notable difference is that while SOX2 is expressed in adult ENS glial cells, glial cells differentiated within the adult DRGs (Schwann cells and satellite glial cells) do not express SOX2 [40]. Interestingly, in both cases, adult glial cells express SOX10 (Fig. 3D; [40]. Thus, although the expression profile of SOX10 is consistent in the adult ENS and DRG glial cells, the expression profile of SOX2 is different. Taken together, our studies uncover a distinct profile of SOX2 within a nervous system lineage.

### Possible Functional Roles for SOX2 in the ENS

In general, the SOXB1 family members (SOX1, SOX2, SOX3) and SOXE family members (SOX8, SOX9, SOX10) are thought to have distinct functions [23]. For example, SOXB1 proteins are expressed in largely overlapping patterns in the CNS [30] and function as neural competence factors and are required for neural stem cell maintenance [34, 41, 42]. SOXE proteins are also expressed in overlapping patterns in the CNS, just prior to the onset of gliogenesis, initially in the presence of SOXB1 proteins and are required for gliogenesis [43, 44]. In different regions of the nervous system (CNS vs. PNS), however, the same SOX proteins are thought to have different functions [23]. For example, in the PNS, SOXE proteins are expressed in migrating neural crest progenitor cells where they function to maintain pluripotency and suppresses neuronal differentiation [8, 35, 41, 45]. The role in maintaining pluripotency is therefore similar in SOXB1 proteins in the CNS and SOXE proteins in the PNS. Our data suggest that SOXB1 proteins may play similar roles in progenitors in the CNS and PNS, as SOX2 is expressed in both CNS and ENS progenitors. Moreover, expression of SOX3 in the developing ENS (SOX1 is not apparently expressed in the ENS; data not shown) suggests that a possible role for SOXB1 proteins in the ENS may extend beyond SOX2.

It has been proposed that SOXB1 proteins and SOXE proteins have opposing functions within NCCs [46]. This point is illustrated by the fact that overexpression of SOX2 blocks NCC formation [39], whereas overexpression of SOX9 leads to overproduction of premigratory NCCs [47]. At these time points, SOX2 and SOX10 have mutually exclusive expression profiles, with SOX2 downregulated in SOX10-expressing premigratory and early migratory NCCs. At later time points, when SOX2 and SOX10 are coexpressed in ENS progenitors and in the glial cell lineage, it is possible that these two proteins are no longer acting antagonistically but rather have similar or even cooperative functions.

*Sox10*-deficient mice have a complete absence of ENS in the gut due to extensive cell death within the vagal NCCs [11, 48–50], and *Sox10*^[+/−]^ mice exhibit aganglionosis of the distal colon, due to loss of ENS progenitors [45, 51]. Despite overlapping expression of SOX2 and SOX10, it is clear that SOX2 cannot compensate for loss of SOX10 during these early stages of ENS development. No functional requirement for SOX2 in ENS development has been revealed using mouse genetic techniques, perhaps confounded by the fact that although *Sox2* heterozygous mice (such as *Sox2*^β*geo*/+^) are viable and have an apparently normal ENS (data not shown and supporting information Fig. 3), *Sox2*^−/−^ embryos die at implantation stages [24]. Further study will require the use of *Sox2* hypomorphic mutations [37] or conditional *Sox2* mutations [52]. Finally, although *Sox10*^[+/−]^ mice do not have obvious defects in gliogenesis, *Sox10*^[+/−]^;*Sox8*^[+/−]^ mice exhibit apparent reductions in glial cell number [45]. Whether glial cell differentiation phenotypes would be compounded by additional mutations in *Sox2* would be of great interest.

### SOX2 As a New Tool to Identify ENS Progenitors

Critical first steps in developing human cell replacement therapies for HSCR are to establish methods to identify and characterize ENS progenitors, and to use animal models to study the potential of these cells on transplantation. A number of techniques have been developed to isolate ENS progenitors from murine or human sources, including selection based on cell surface marker expression, cell culture techniques that favor growth of progenitors, and selection on the basis of proliferative capacity [2, 17]. A limitation to these approaches is that cells are available in small numbers, and can represent mixed cell populations, containing differentiated cells along with progenitors. Still other techniques aim to use embryonic stem cells or neural stem cells as ENS progenitors [2, 17, 53]. Although these techniques have the capacity to grow cells in large numbers, their usefulness depends on the ability to purify cells of specific ENS progenitor potential, a technique that has yet to be established.

The approach we describe here attempts to circumvent previous limitations. Having identified SOX2 as a marker of enteric progenitor cells, we combined genetic and cell culture techniques to enable a *Sox2*-expressing population to be selected. Such cells can be expanded and passaged in culture for months, and thus provide ENS progenitors in large numbers. Selection on the basis of *Sox2* expression has the added benefit of limiting the number of neurons present in the population, thus reducing the heterogeneity of the population. The usefulness of this strategy in obtaining cells with appropriate progenitor properties is demonstrated by the fact that cells transplanted into the gut can migrate, differentiate, and establish close associations with the endogenous ENS. Moreover, such cells are capable of colonizing aganglionic gut regions. Recent experiments using a similar approach to select embryonic cells on the basis of *Sox10* expression has also successfully enriched for cells with migratory and differentiation potential [54]. We suggest that these techniques will facilitate the development of cell replacement therapies in animal models, a critical first step in developing methods to be used in treating human HSCR patients.

## CONCLUSION

We have identified SOX2 as a novel marker of enteric progenitor cells and glial cells, a finding that will facilitate the study of ENS progenitors. In addition, we have used a selection technique to enrich for SOX2-expressing ENS progenitors among cultured gut cells. This method enables ENS progenitors to be obtained in the large numbers required for studying their properties following transplantation, and to develop cell replacement therapies using animal models. Our experiments show that *Sox2*^β*geo*^ cells transplanted into embryonic gut can migrate extensively, differentiate appropriately, and appear to be integrated into the endogenous ENS ganglia. Thus, *Sox2*^β*geo*^ cells also exhibit key properties of ENS progenitors, further supporting their potential use in modeling cell replacement therapies in animal models, with eventual implications on treatment of HSCR.

## References

[1] Li L, Clevers H (2010). Coexistence of quiescent and active adult stem cells in mammals. Science.

[2] Heanue TA, Pachnis V (2007). Enteric nervous system development and Hirschsprung's disease: Advances in genetic and stem cell studies. Nat Rev Neurosci.

[3] Newgreen D, Young HM (2002). Enteric nervous system: Development and developmental disturbances—Part 2. Pediatr Dev Pathol.

[4] Newgreen D, Young HM (2002). Enteric nervous system: Development and developmental disturbances—Part 1. Pediatr Dev Pathol.

[5] Burzynski G, Shepherd IT, Enomoto H (2009). Genetic model system studies of the development of the enteric nervous system, gut motility and Hirschsprung's disease. Neurogastroenterol Motil.

[6] Baillie CT, Kenny SE, Rintala RJ (1999). Long-term outcome and colonic motility after the Duhamel procedure for Hirschsprung's disease. J Pediatr Surg.

[7] Menezes M, Corbally M, Puri P (2006). Long-term results of bowel function after treatment for Hirschsprung's disease: A 29-year review. Pediatr Surg Int.

[8] Bondurand N, Natarajan D, Barlow A (2006). Maintenance of mammalian enteric nervous system progenitors by SOX10 and endothelin 3 signalling. Development.

[9] Danielian PS, Muccino D, Rowitch DH (1998). Modification of gene activity in mouse embryos in utero by a tamoxifen-inducible form of Cre recombinase. Curr Biol.

[10] Srinivas S, Watanabe T, Lin CS (2001). Cre reporter strains produced by targeted insertion of EYFP and ECFP into the ROSA26 locus. BMC Dev Biol.

[11] Southard-Smith EM, Kos L, Pavan WJ (1998). Sox10 mutation disrupts neural crest development in Dom Hirschsprung mouse model. Nat Genet.

[12] Young HM, Bergner AJ, Anderson RB (2004). Dynamics of neural crest-derived cell migration in the embryonic mouse gut. Dev Biol.

[13] Young HM, Turner KN, Bergner AJ (2005). The location and phenotype of proliferating neural-crest-derived cells in the developing mouse gut. Cell Tissue Res.

[14] Pham TD, Gershon MD, Rothman TP (1991). Time of origin of neurons in the murine enteric nervous system: Sequence in relation to phenotype. J Comp Neurol.

[15] Rothman TP, Tennyson VM, Gershon MD (1986). Colonization of the bowel by the precursors of enteric glia: Studies of normal and congenitally aganglionic mutant mice. J Comp Neurol.

[16] Young HM, Bergner AJ, Muller T (2003). Acquisition of neuronal and glial markers by neural crest-derived cells in the mouse intestine. J Comp Neurol.

[17] Schafer KH, Micci MA, Pasricha PJ (2009). Neural stem cell transplantation in the enteric nervous system: Roadmaps and roadblocks. Neurogastroenterol Motil.

[18] Almond S, Lindley RM, Kenny SE (2007). Characterisation and transplantation of enteric nervous system progenitor cells. Gut.

[19] Bogni S, Trainor P, Natarajan D (2008). Non-cell-autonomous effects of Ret deletion in early enteric neurogenesis. Development.

[20] Natarajan D, Grigoriou M, Marcos-Gutierrez CV (1999). Multipotential progenitors of the mammalian enteric nervous system capable of colonising aganglionic bowel in organ culture. Development.

[21] Heanue TA, Pachnis V (2006). Expression profiling the developing mammalian enteric nervous system identifies marker and candidate Hirschsprung disease genes. Proc Natl Acad Sci USA.

[22] Pevny LH, Nicolis SK (2010). Sox2 roles in neural stem cells. Int J Biochem Cell Biol.

[23] Wegner M, Stolt CC (2005). From stem cells to neurons and glia: A Soxist's view of neural development. Trends Neurosci.

[24] Avilion AA, Nicolis SK, Pevny LH (2003). Multipotent cell lineages in early mouse development depend on SOX2 function. Genes Dev.

[25] Belyaev NN, Brown DE, Diaz AI (2010). Induction of an IL7-R(+)c-Kit(hi) myelolymphoid progenitor critically dependent on IFN-gamma signaling during acute malaria. Nat Immunol.

[26] Bondurand N, Natarajan D, Thapar N (2003). Neuron and glia generating progenitors of the mammalian enteric nervous system isolated from foetal and postnatal gut cultures. Development.

[27] Taraviras S, Marcos-Gutierrez CV, Durbec P (1999). Signalling by the RET receptor tyrosine kinase and its role in the development of the mammalian enteric nervous system. Development.

[28] Denaxa M, Sharpe PT, Pachnis V (2009). The LIM homeodomain transcription factors Lhx6 and Lhx7 are key regulators of mammalian dentition. Dev Biol.

[29] Druckenbrod NR, Epstein ML (2005). The pattern of neural crest advance in the cecum and colon. Dev Biol.

[30] Wood HB, Episkopou V (1999). Comparative expression of the mouse Sox1, Sox2 and Sox3 genes from pre-gastrulation to early somite stages. Mech Dev.

[31] Le Douarin NM, Teillet MA (1974). Experimental analysis of the migration and differentiation of neuroblasts of the autonomic nervous system and of neurectodermal mesenchymal derivatives, using a biological cell marking technique. Dev Biol.

[32] Kuhlbrodt K, Herbarth B, Sock E (1998). Sox10, a novel transcriptional modulator in glial cells. J Neurosci.

[33] Burns AJ (2005). Migration of neural crest-derived enteric nervous system precursor cells to and within the gastrointestinal tract. Int J Dev Biol.

[34] Bylund M, Andersson E, Novitch BG (2003). Vertebrate neurogenesis is counteracted by Sox1–3 activity. Nat Neurosci.

[35] Kim J, Lo L, Dormand E (2003). SOX10 maintains multipotency and inhibits neuronal differentiation of neural crest stem cells. Neuron.

[36] Liu MT, Kuan YH, Wang J (2009). 5-HT4 receptor-mediated neuroprotection and neurogenesis in the enteric nervous system of adult mice. J Neurosci.

[37] Ferri AL, Cavallaro M, Braida D (2004). Sox2 deficiency causes neurodegeneration and impaired neurogenesis in the adult mouse brain. Development.

[38] Zappone MV, Galli R, Catena R (2000). Sox2 regulatory sequences direct expression of a (beta)-geo transgene to telencephalic neural stem cells and precursors of the mouse embryo, revealing regionalization of gene expression in CNS stem cells. Development.

[39] Wakamatsu Y, Endo Y, Osumi N (2004). Multiple roles of Sox2, an HMG-box transcription factor in avian neural crest development. Dev Dyn.

[40] Aquino JB, Hjerling-Leffler J, Koltzenburg M (2006). In vitro and in vivo differentiation of boundary cap neural crest stem cells into mature Schwann cells. Exp Neurol.

[41] Graham V, Khudyakov J, Ellis P (2003). SOX2 functions to maintain neural progenitor identity. Neuron.

[42] Kishi M, Mizuseki K, Sasai N (2000). Requirement of Sox2-mediated signaling for differentiation of early *Xenopus* neuroectoderm. Development.

[43] Stolt CC, Lommes P, Sock E (2003). The Sox9 transcription factor determines glial fate choice in the developing spinal cord. Genes Dev.

[44] Stolt CC, Schmitt S, Lommes P (2005). Impact of transcription factor Sox8 on oligodendrocyte specification in the mouse embryonic spinal cord. Dev Biol.

[45] Maka M, Stolt CC, Wegner M (2005). Identification of Sox8 as a modifier gene in a mouse model of Hirschsprung disease reveals underlying molecular defect. Dev Biol.

[46] Guth SI, Wegner M (2008). Having it both ways: Sox protein function between conservation and innovation. Cell Mol Life Sci.

[47] Cheung M, Briscoe J (2003). Neural crest development is regulated by the transcription factor Sox9. Development.

[48] Britsch S, Goerich DE, Riethmacher D (2001). The transcription factor Sox10 is a key regulator of peripheral glial development. Genes Dev.

[49] Herbarth B, Pingault V, Bondurand N (1998). Mutation of the Sry-related Sox10 gene in Dominant megacolon, a mouse model for human Hirschsprung disease. Proc Natl Acad Sci USA.

[50] Kapur RP (2000). Colonization of the murine hindgut by sacral crest-derived neural precursors: Experimental support for an evolutionarily conserved model. Dev Biol.

[51] Paratore C, Eichenberger C, Suter U (2002). Sox10 haploinsufficiency affects maintenance of progenitor cells in a mouse model of Hirschsprung disease. Hum Mol Genet.

[52] Taranova OV, Magness ST, Fagan BM (2006). SOX2 is a dose-dependent regulator of retinal neural progenitor competence. Genes Dev.

[53] Hotta R, Pepdjonovic L, Anderson RB (2009). Small-molecule induction of neural crest-like cells derived from human neural progenitors. STEM CELLS.

[54] Kawaguchi J, Nichols J (2010). Isolation and propagation of enteric neural crest progenitor cells from mouse embryonic stem cells and embryos. Development.

