# Supplementary figures and images for: Prospective Identification and Isolation of Enteric Nervous System Progenitors Using Sox2
Source: Stem Cells. 2010 Oct 16;29(1):128–40. doi: 10.1002/stem.557 (PMC3059409; doi:10.1002/stem.557)

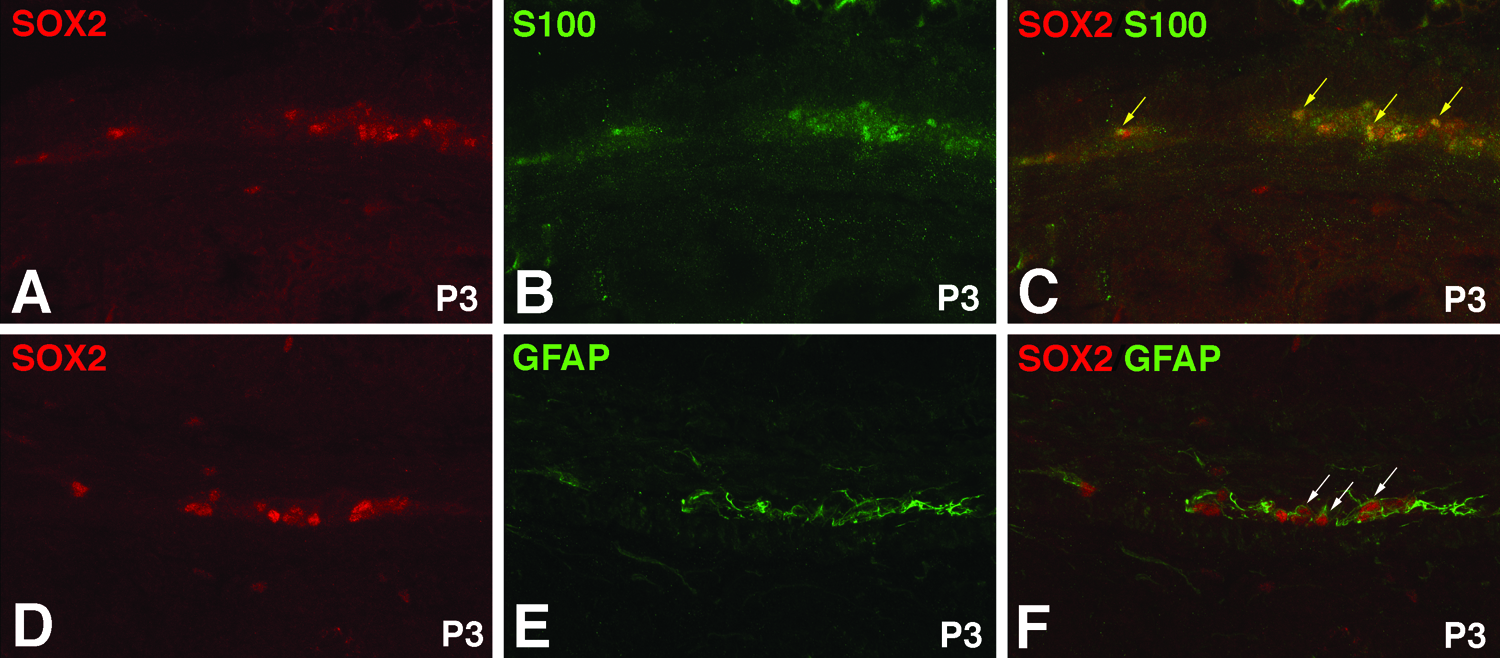

Supplement: Supplementary file 1 [file stem0029-0128-SD1.tif]

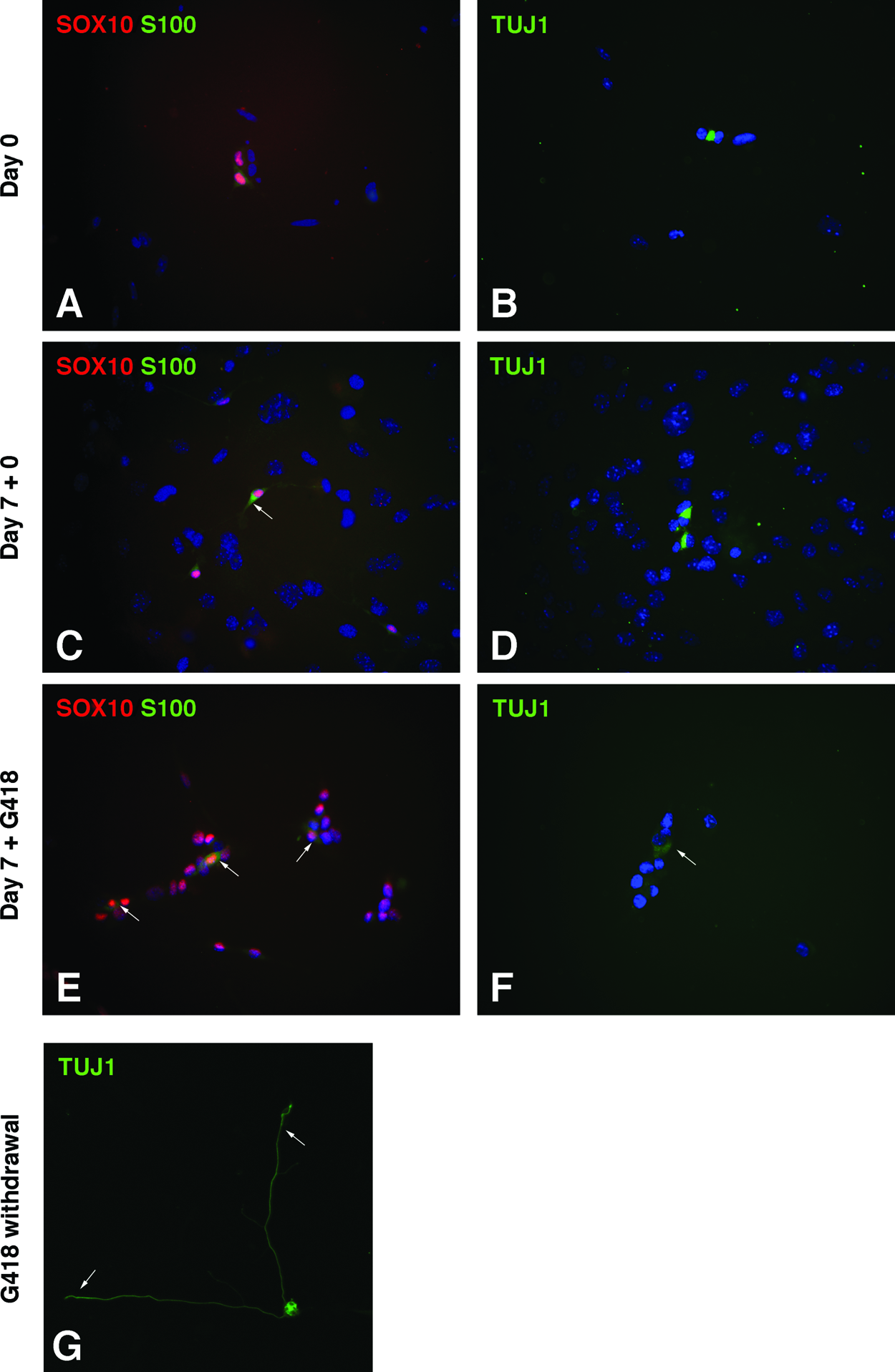

Supplement: Supplementary file 2 [file stem0029-0128-SD2.tif]

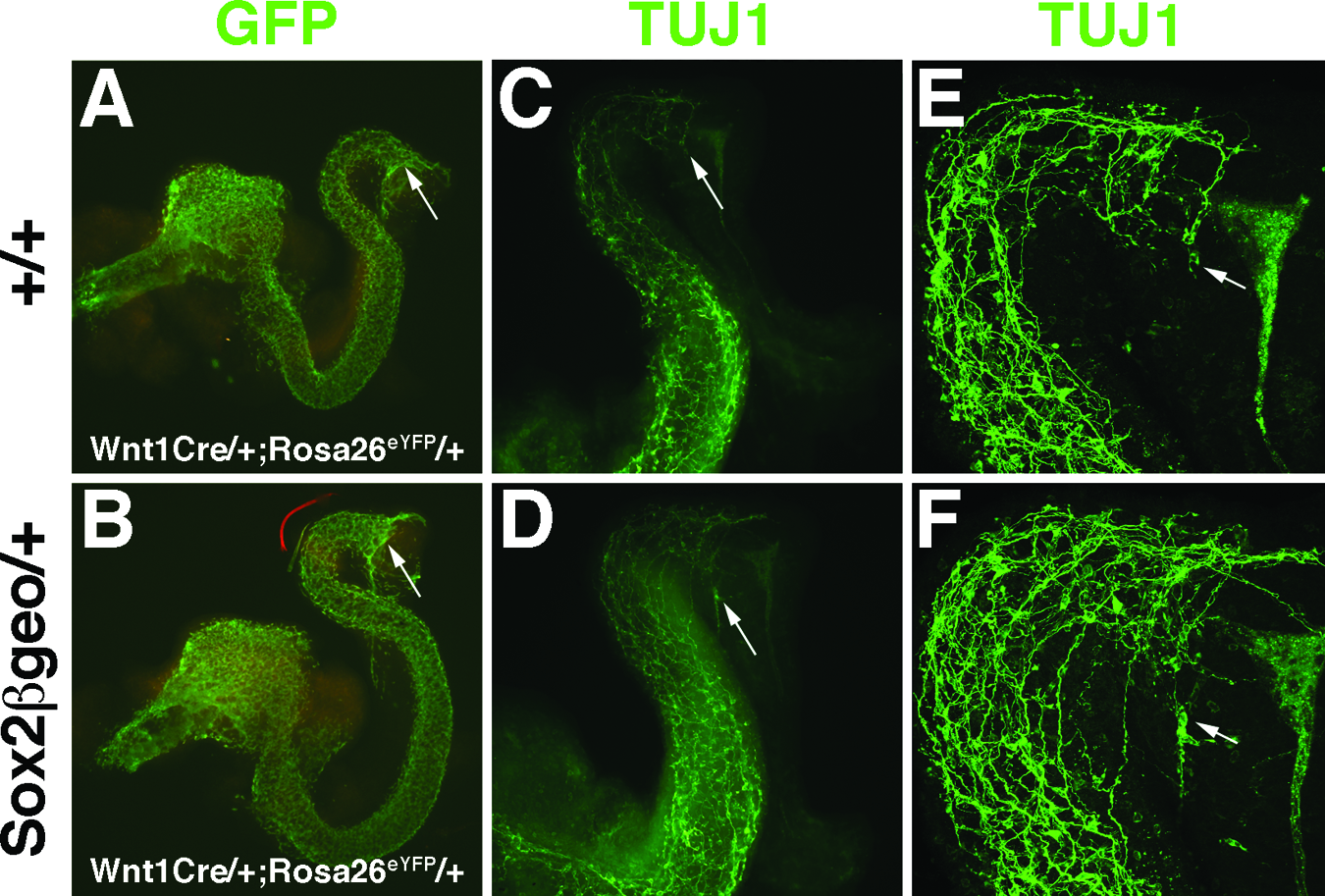

Supplement: Supplementary file 3 [file stem0029-0128-SD3.tif]
